# S125. THE DYNAMIC RELATIONSHIP BETWEEN INSIGHT AND SUICIDAL BEHAVIOUR IN FIRST EPISODE PSYCHOSIS PATIENTS OVER 3-YEAR FOLLOW-UP

**DOI:** 10.1093/schbul/sby018.912

**Published:** 2018-04-01

**Authors:** Rosa Ayesa-Arriola, Jose Maria Pelayo-Teran, Javier-David Lopez-Morinigo, Manuel Canal-Rivero, Esther Setien-Suero, Manuel J Cuesta, Anthony David, Benedicto Crespo-Facorro

**Affiliations:** 1 Marqués de Valdecilla University Hospital, IDIVAL, School of Medicine, University of Cantabria; 2 Servicio de Psiquiatría, Hospital El Bierzo, SACYL, Ponferrada, Castilla y León; 3 Institute of Psychiatry, Psychology and Neuroscience, King’s College London; 4 University Hospital Virgen del Rocio, Autonomus University of Barcelona; 5 University Hospital Marques de Valdecilla -IDIVAL, University of Cantabria, CIBERSAM; 6 Complejo Hospitalario de Navarra, IdiSNA, Navarra Institute for Health Research

## Abstract

**Background:**

Studies have established the high risk of suicide in first episode psychosis (FEP). Between 15%-26% of FEP patients attempt suicide at least once before their first contact with psychiatric services and 2–5% die from suicide. Also, many patients with schizophrenia spectrum disorders lack insight into having a mental disorder. However, the relationship between insight changes and suicidal behaviour in FEP remains poorly understood.

**Methods:**

Information about suicidal behaviour was available on a cohort of 397 FEP patients. Three dimensions of insight (into mental illness, the need for treatment, and the social consequences) were measured at: baseline, 1 and 3 years after the initiation of treatment. Survival analyses examined time to suicidal behavior in relation to i) insight at baseline, ii) the closest insight measure to the suicide attempt, and iii) changes in insight during the follow-up.

**Results:**

No associations were found between baseline insight dimensions and time to suicidal behaviour. However, poor insight at the evaluation closest to the suicide attempt was associated with an increased risk of suicide. Stability of insight did not affect the risk of suicidal behaviour, while changes in either direction were linked with an increased risk of suicidal behaviour, particularly worsening insight.

**Discussion:**

Insight in psychosis is a dynamic concept and we demonstrated the relationship between insight and suicide risk to be equally dynamic. Poor insight seems to increase the risk, especially when insight levels change. Repeated insight assessment to detect change from early psychosis may play a role in suicide prevention.

